# Simultaneous Measurement of Amino Acid Enantiomers in Aged Mouse Brain Samples by LC/MS/MS Combined with Derivatization Using *N*^α^-(5-Fluoro-2,4-dinitrophenyl)-l-leucinamide (l-FDLA)

**DOI:** 10.3390/metabo11010057

**Published:** 2021-01-15

**Authors:** Taiji Yamamoto, Keisuke Yaku, Takashi Nakagawa

**Affiliations:** 1 Department of Molecular and Medical Pharmacology, Faculty of Medicine, University of Toyama, Toyama 930-0194, Japan; s1550102@ems.u-toyama.ac.jp (T.Y.); yaku@med.u-toyama.ac.jp (K.Y.); 2 Research Center for Pre-Disease Science, University of Toyama, Toyama 930-0194, Japan

**Keywords:** d-amino acid, LC/MS/MS, l-FDLA, aging, brain

## Abstract

d-amino acids have distinct roles from their l-enantiomer. In particular, some d-amino acids function as agonists or antagonists of neuronal receptors and are involved in higher brain functions. Thus, it is important to precisely measure the levels of these amino acid enantiomers in cells and tissues. Various quantification methods have been developed for measurements of chiral amino acids. However, each method has advantages and disadvantages. Additionally, measuring the amino acid enantiomers in crude biological samples requires a higher selectivity. In this study, we developed a quantification method for amino acid enantiomers using derivatization with *N*^α^-(5-Fluoro-2,4-dinitrophenyl)-l-leucinamide (l-FDLA) followed by liquid chromatography–tandem mass spectrometry (LC/MS/MS) with a conventional reversed-phase column. We simultaneously identified 10 chiral amino acids. Furthermore, we applied this method to investigate murine tissue samples and examined the effect of aging on the amino acid levels in aged brain regions. We found that aging decreased the levels of both d-serine and d-aspartate in the hippocampus. In addition, d-Phenylalanine in the thalamus significantly increased with age. In conclusion, our method is suitable for the quantification of the d-amino acids in crude biological samples and may contribute to elucidating the biological roles of chiral amino acids.

## 1. Introduction

Amino acids are pivotal nutrients which serve as energy sources and building blocks for organisms. Twenty amino acids make up the mammalian proteins. They all (except glycine) have one or two chiral centers where four different functional groups or atoms are attached to the same carbon [1]. Therefore, each amino acid has two enantiomeric forms, denoted l- and d-amino acids. In mammals, although l-amino acids predominate, the d-enantiomers also exist and have biological functions in certain tissues such as the brain [2]. In particular, d-serine (d-Ser) and d-aspartate (d-Asp) are relatively abundant in the brain and have distinct roles from their l-enantiomers [3,4,5]. d-Asps were the first free d-amino acids identified in mammals, including humans, and are a precursor for *N*-methyl-d-aspartate (NMDA) [4,6,7,8]. d-Asp is abundant in the neonatal phase and its amount decreases after birth as the expression of d-aspartate oxidase increases [8,9,10]. d-Ser is another major free d-amino acid produced from l-Ser by serine racemase and is a co-agonist of the NMDA receptor that regulates neural functions [5]. Additionally, d-amino acids act on endocrine systems, thus might be important to maintain homeostasis [4,11]. d-Asp stimulates the production of several hormones in the pituitary and hypothalamus, and also promotes the production of testosterone in the testis [12,13,14]. Accordingly, the functions of d-amino acids have been increasingly investigated, and quantification methods have been developed to investigate their biological roles.

d-amino acid quantification methods require high sensitivity and selectivity because the abundance of d-amino acids in crude biological samples is low relative to l-amino acids. Numerous l- and d-amino acid quantification strategies have been reported. One of the approaches to detect d-amino acids is to employ enzymes that process d-amino acids but not l-amino acids [15]. d-amino acid oxidase (DAO) is often used for enzymatic detection of d-amino acids [15,16,17]. It converts basic and neutral d-amino acids to α-keto acids in the presence of flavin adenine dinucleotide. Although enzymatic methods using DAO are relatively easy and cheap, it is difficult to distinguish each d-amino acid in crude biological samples. Thus, quantifying d-amino acids in crude biological samples requires analytical separation techniques. To achieve a chiral resolution of amino acid enantiomers, high-performance liquid chromatography (HPLC) is frequently employed [18,19,20]. However, conventional octadecylsilane (ODS) columns cannot separate free d- and l-amino acids because their retention times are identical [21]. Thus, chiral columns have been used for this purpose. They are packed with a chiral stationary phase that can separate the amino acid enantiomers [22,23]. However, these chiral columns have restrictions regarding mobile phase solvents and their durability is lower than that of conventional ODS columns [24]. Additionally, the detection of low amounts of d-amino acids requires baseline separation. Thus, liquid chromatography–mass spectrometry (LC/MS) is often employed, however several chiral columns are incompatible with LC/MS due to solvent restriction.

Another method for the indirect chiral resolution of amino acid enantiomers is chemical derivatization. Chemical derivatization allows separation on conventional ODS columns [25]. It also allows taking advantage of increased signals and low background noise to detect low amounts of d-amino acids. Several chiral derivatizing reagents have been developed. In 1984, Marfey developed the *N*^α^-(5-Fluoro-2,4-dinitrophenyl)-l-alaninamide (l-FDAA, also named Marfey’s reagent) enabling the indirect chiral resolution of amino acid enantiomers and successfully separated the diastereomeric l- and d-amino acids by HPLC [26]. This Marfey’s reagent is also compatible with LC/MS analysis [27]. Fujii et al. reported an advanced Marfey’s reagent, *N*^α^-(5-Fluoro-2,4-dinitrophenyl)-l-leucinamide (l-FDLA) [28]. They showed that l-FDLA exhibited a higher sensitivity and separation for derivatized chiral amino acids compared with l-FDAA.

In this study, we used l-FDLA derivatization combined with LC/MS/MS to simultaneously quantify l- and d-amino acids in crude biological samples. Furthermore, we applied this method to examine the effect of aging on the amino acid levels in aged brain regions. We found that aging decreased the levels of both d-Ser and d-Asp in the hippocampus. In addition, d-Phenylalanine (d-Phe) in the aged thalamus significantly increased.

## 2. Results

### 2.1. Optimization of the Multiple Reaction Monitoring (MRM) Settings of LC/MS/MS for l-FDLA Derivatized Amino Acids

**Figure 1:**
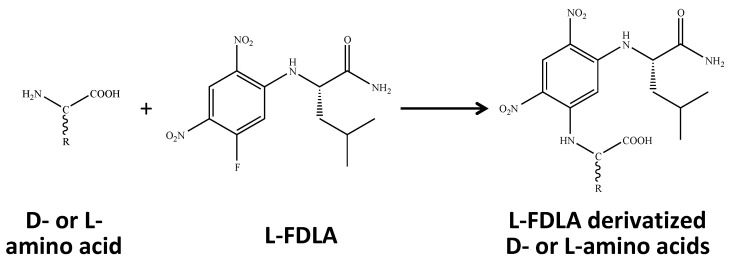
Scheme of derivatization for amino acid enantiomers by *N*^α^-(5-Fluoro-2,4-dinitrophenyl)-l-leucinamide (l-FDLA). l-FDLA-derivatized amino acids become diastereomeric and can be separated by a conventional reversed-phase high-performance liquid chromatography (HPLC) column.

For this study, we prepared 10 commercially available d- and l-amino acids, including alanine (Ala), asparagine (Asn), aspartate (Asp), leucine (Leu), methionine (Met), proline (Pro), serine (Ser), glutamine (Gln), glutamate (Glu), and phenylalanine (Phe). Standard l-amino acids were derivatized with l-FDLA as described in the method section. The derivatization added approximately 294 Da to the molecular weight of the l-amino acid (Figure 1). We performed mass spectrometry in electrospray ionization (ESI) positive ion mode and determined the MRM settings using the direct infusion method. For all metabolites, we selected [M+H]^+^ as the precursor ion and confirmed the intensity using the selected ion monitoring (SIM) mode. We chose the N_2_ gas collision-induced fragment ion based on the criteria that it had the highest intensity. We optimized the fragmentor voltage and collision energy to achieve maximum MS intensity. We also confirmed the MRM settings for derivatized d-amino acid compounds, but all parameters were similar to l-amino acids derivatized with l-FDLA.

### 2.2. Detection of Derivatized Standard Compounds by LC/MS/MS

**Figure 2:**
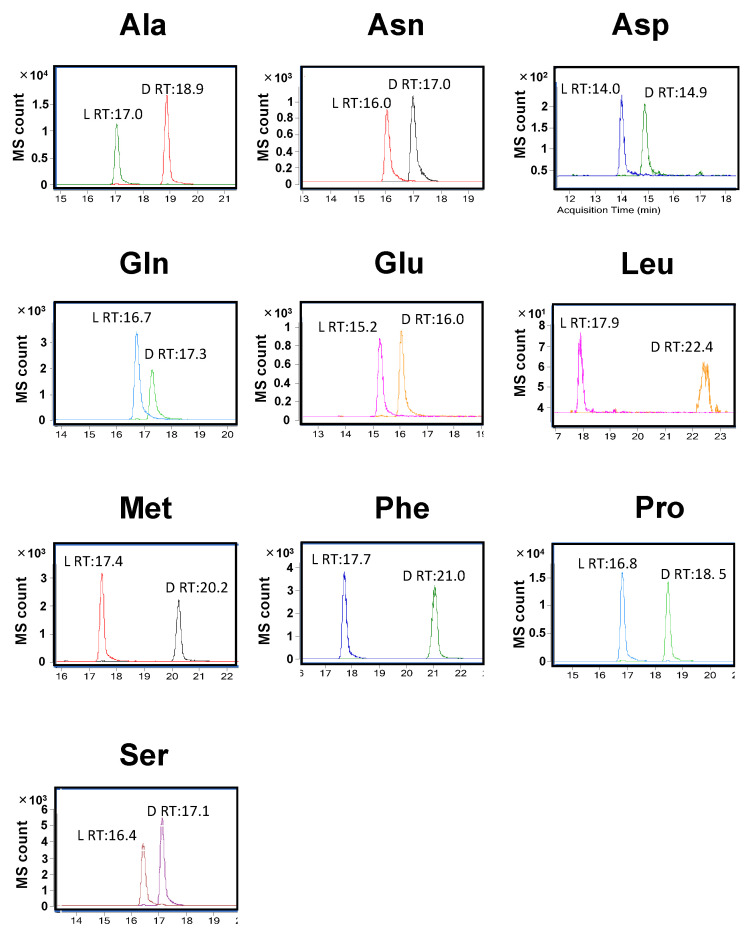
Chromatograms of standard compounds of derivatized l- and d-amino acids. Standard l- and d-amino acids were derivatized with l-FDLA and analyzed by LC/MS/MS. Representative MRM chromatograms of derivatized l- and d-amino acids are presented as merged to confirm the drift of retention time. In each panel, relative ion abundance (MS counts) versus retention time (min) are shown.

Conventional reversed-phase HPLC columns cannot separate underivatized amino acid enantiomers. Thus, chiral or enantioselective derivatization is essential for the separation of d- and l-amino acid enantiomers. Besides, it is crucial to accurately identify d- and l-amino acids in crude biological samples. However, the molecular weight difference between Gln and Glu, or Asn and Asp, is approximately 1 Da, and the mass accuracy of a typical tandem mass spectrometer is around 0.5 Da. Thus, they are difficult to distinguish using a typical tandem mass spectrometer without performing HPLC column separation due to the detectable decimal mass limit, and the presence of natural isotopes, such as ^13^C. For the separation, we employed an MG3 column, a conventional reversed-phase ODS column, and performed chromatographic analysis using gradient elution with 5 mM ammonium formate in water as mobile phase A and 100% of methanol as mobile phase B. The gradient elution went from 80% to 20% of mobile phase A. Using this analytical setting, we could distinguish the l- and d-enantiomers of all amino acids we tested (Figure 2). In all cases, l-enantiomers were eluted earlier than d-enantiomers. The retention time (RT) of l- and d-enantiomers differed by more than 0.6 min. The RT of l-Glu and d-Glu were approximately 15.2 and 16.0 min, respectively. Those of l-Gln and d-Gln were approximately 16.7 and 17.3 min, respectively (Table 1 and Figure 2). Furthermore, we separated and distinguished Asp and Asn enantiomers (Table 1 and Figure 2). Using this method, we chromatographically separated most of the amino acid enantiomers.

### 2.3. Detection of Amino Acid Enantiomers in Biological Samples

**Figure 3:**
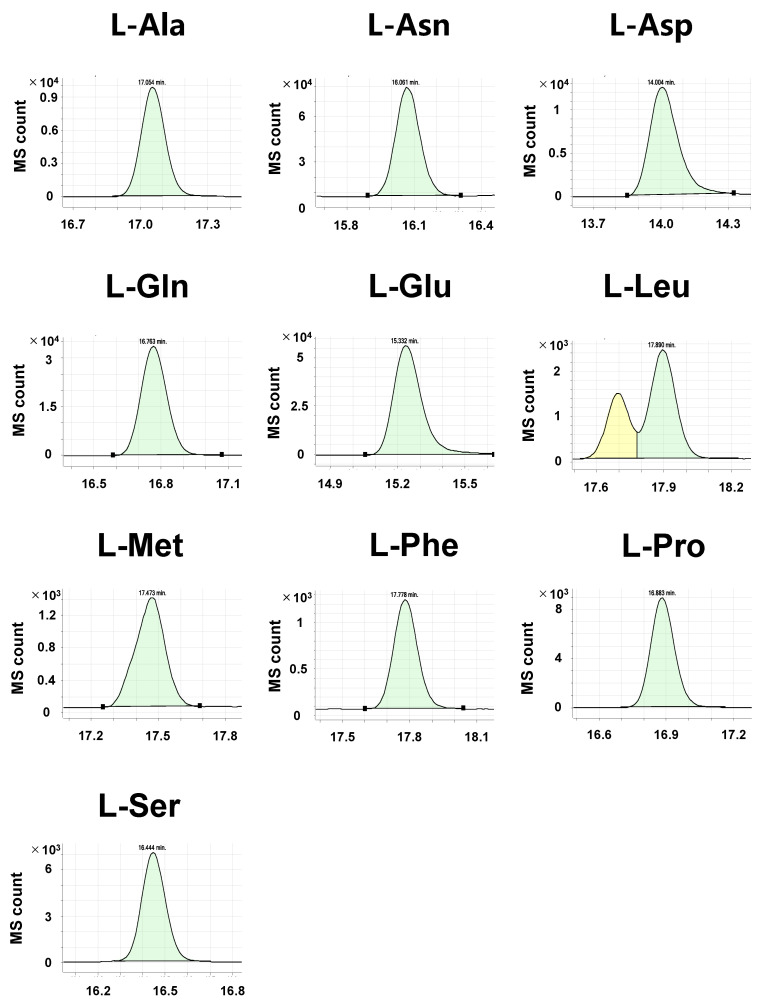
Chromatograms of derivatized l-amino acids in the murine cortex samples. Tissue samples were extracted from the cortices of 3-month-old mice. Representative MRM chromatograms of derivatized l-amino acids are shown. In each panel, relative ion abundance (MS counts) versus retention time (min) are shown. Integrated MS count (green) represents the amount of target molecules.

**Figure 4:**
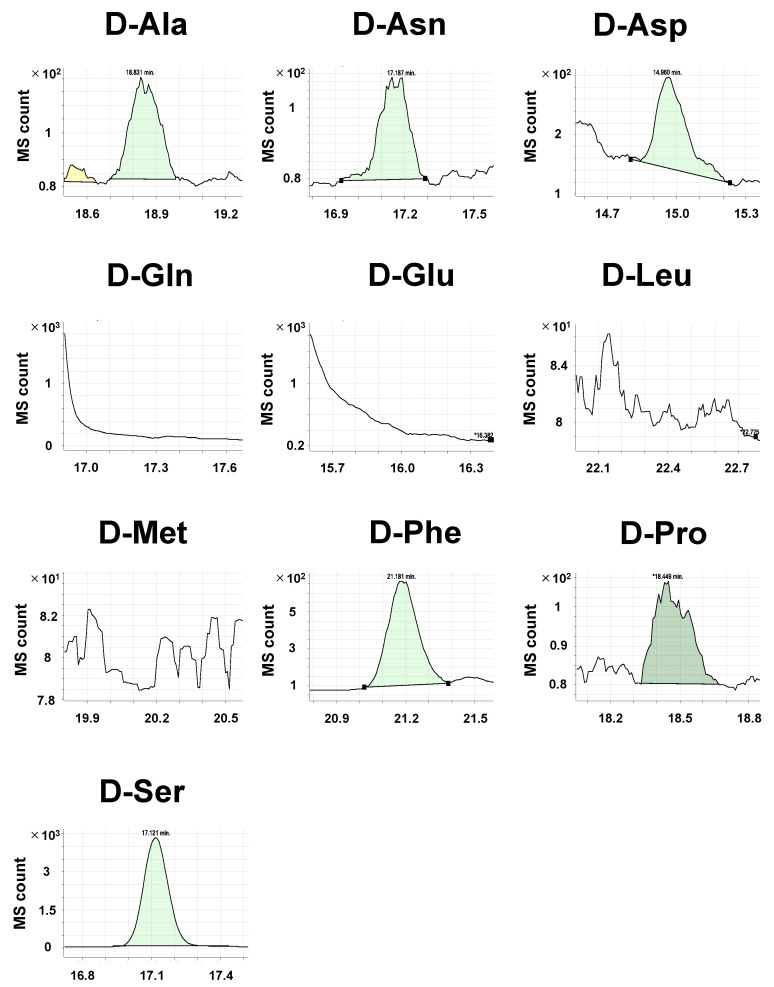
Chromatograms of derivatized d-amino acids in the murine cortex samples. Tissue samples were extracted from the cortices of 3-month-old mice. Representative MRM chromatograms of derivatized d-amino acids are shown. In each panel, relative ion abundance (MS counts) versus retention time (min) are shown. Integrated MS count (green) represents the amount of target molecules.

Compared with the pure standard compounds, crude biological samples, such as cell lysate and tissue homogenate, exhibit complex chromatograms. Some d-amino acids act as neurotransmitters and are abundant in brain tissues [29,30]. Thus, we attempted to detect amino acid enantiomers in murine brain cortex samples to validate our method. After euthanization, C57BL/6N mice brains were immediately collected and the cortex was separated. In these samples, we detected all the l-amino acids, but l-Leu had a bimodal peak at the expected retention time (Figure 3). In the brain, d-Ser is reportedly the d-amino acid with the highest concentration [7,31,32]. Accordingly, we found a prominent peak corresponding to d-Ser (Figure 4). We also observed peaks for d-Asn, d-Asp, d-Phe, d-Ala, and d-Pro. Although the peak of l-Glu was evident, that of d-Glu was difficult to detect. Similarly, d-Gln, d-Leu, and d-Met were impossible to detect.

### 2.4. Validation of the Absolute Quantification of Amino Acid Enantiomers

**Figure 5:**
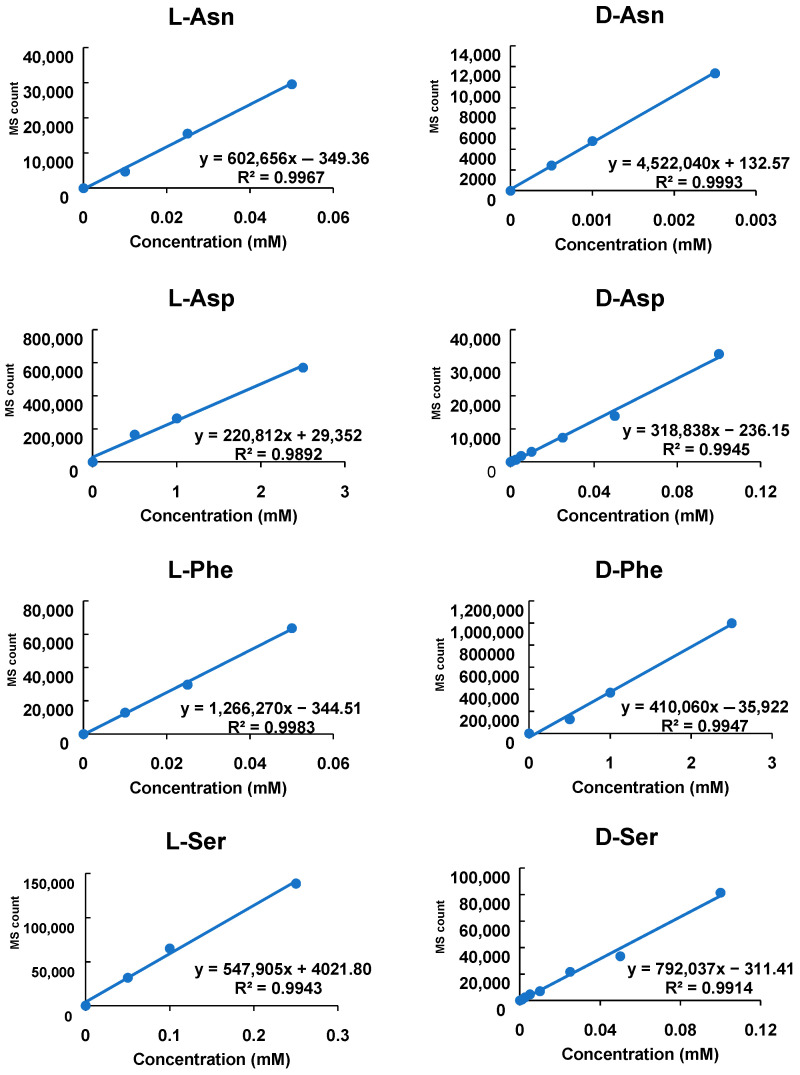
Standard curves of derivatized l- and d-amino acids. Trends of MS count to amino acid concentrations were calculated by measuring the several standard solutions. The *X*-axis represents concentrations of amino acids before being derivatized, and the *Y*-axis represents the integrated sum of the peak area from each chromatogram. The equations and R^2^ values of each plot are shown.

To quantify the levels of d- and l-Asp, Asn, Ser, and Phe absolutely, we measured various concentrations of standard compounds using our method. We confirmed that the plots were linear within the concentrations that were tested (Figure 5). All tested compounds had R^2^ values > 0.98. The equation coefficient in d-enantiomer is higher in Asn, Asp, and Ser, but lower in Phe. Although the sensitivity of LC/MS/MS was slightly lower for d-enantiomers, our method is suitable for the absolute quantification of both d- and l-enantiomers.

### 2.5. Changes of Amino Acid Enantiomers in Aged Brains

d-Ser is an endogenous ligand of the NMDA receptor and its decline is associated with synaptic plasticity and memory deficits in normal aging [33,34,35]. Indeed, it has been reported d-Ser levels decline with age in the hippocampus [33]. However, it is still unclear whether levels of other d-amino acid enantiomers decrease similarly. Here we applied our method to compare the levels of enantiomers of Asp, Asn, Ser, and Phe in the cerebellum, cortex, hippocampus, and thalamus of young and old animals. As previously reported, the levels of d-Ser decreased in the hippocampus with age (Table 2).

However, we observed no changes in the cerebellum, cortex, and thalamus. Additionally, the levels of d-Asp significantly declined in the aged hippocampus. Regarding l-enantiomers, l-Asn, l-Asp, and l-Ser also declined in the hippocampus with age. In the cerebellum and cortex, the levels of all of amino acid enantiomers were the same between young and aged mice. Interestingly, d-Phe, but not l-Phe, significantly increased with age in the thalamus. Thus, we established that our method was applicable to quantify amino acid enantiomers in crude biological samples.

## 3. Discussion

In this study, we have developed a new absolute quantification method for amino acid enantiomers and validated its application to murine tissue samples. Many analytical methods have been reported for measurements of l-and d-amino acids. Separation before detection is essential for the accurate identification of chiral amino acids. Previously, liquid chromatographic methods using pre-column derivatization with chiral reagents have been developed for the indirect enantioseparation of d- and l-amino acids. These methods combined HPLC separation with diastereomerization using reagents such as l-FDAA [26], 1-(9-Fluorenyl)ethyl chloroformate [36], S-flunoxaprofen [37], or 1-(9-anthryl)-2-propyl chloroformate [38]. Additionally, *o*-phthaldialdehyde has been used in combination with *N*-acetyl-l-cysteine or *N*-*tert*-butyloxycarbonyl-l-cysteine [39,40]. Most of these methods used UV absorbance or fluorescence for the detection. However, these detection methods are less selective than MS detection. Therefore, derivatizing reagents compatible with MS are required for amino acid metabolomics. l-FDLA is a derivative of l-FDAA and gives more robust signals than l-FDAA in LC/MS [28]. However, we did not directly compare the detection sensitivity between l-FDAA- and l-FDLA-derivatized amino acids in this study, and further investigation is necessary to confirm the superiority of our method.

We applied our method to 10 pairs of amino acids. Our method worked for all the amino acids tested in this study when we measured each standard compound. On the other hand, detectable d-amino acids were limited in biological samples. We could not detect d-Gln, d-Glu, d-Leu, and d-Met, while we observed all the l-amino acids in the murine cortex. In particular, we could detect relatively prominent peaks of l-Glu and l-Gln in the cortex sample but failed to detect their d-enantiomers. Previous study also indicated that the ratio of l- and d-Glu and Gln is relatively large compared to other amino acids [41]. Previously, another group tried to map the regional distribution of d-amino acids in the brain, but they also failed to detect d-Asp, d-Ala, and d-Leu in the cerebrum, hypothalamus, and cerebellum in the 6-week-old rats [32]. Therefore, our method is more sensitive and applicable to the biological samples, at least for d-Asp and d-Ala. Regarding d-Ser, they also could determine its concentration in the cerebellum and hippocampus and reported their levels as 0.210 and 0.231 µmol/g tissue, respectively. In this study, we determined the levels of d-Ser in the hippocampus as 0.646 µmol/g tissue using 3-month-old mice. Thus, the sensitivity is almost comparable for d-Ser. Meanwhile, Inoue et al. successfully measured d-Leu in some brain regions including the cerebrum by combining 2D-HPLC with 4-Fluoro-7-nitrobenzofurazan derivatization [42]. Our fault in d-Leu detection may be attributed to ion suppression. Moreover, we got two peaks in l-Leu MRM possibly due to the incomplete separation of Leu and Ile. Therefore, the separation of these amino acids needs to be optimized by trying other columns and/or solvents.

Elucidating the distributions of d-amino acids in various tissues helps to clarify the physiological functions of d-amino acids. In the present study, we applied our method to determine the d-amino acid levels in various brain regions because of the importance of d-Ser and d-Asp in neuronal functions. We confirmed that the levels of both d-Ser and d-Asp decreased in the hippocampus with age. Besides, d-Phe significantly increased with age in the thalamus. In the brain, l-Phe is a competitive antagonist of the NMDA and AMPA receptors [43,44]. d-Phe is also an agonist of the niacin receptor 2 (NIACR2) [45]. However, the biological role of d-Phe is unknown. Thus, investigating the role of d-Phe and NIACR2 in the aged thalamus would of interest. Additionally, d-amino acids also exist in the heart, lungs, kidney, liver, thyroid, pancreas, adrenal gland, testes, and ovaries, but little is known about their importance in these tissues [14,16,46,47,48,49]. Interestingly, levels of d-amino acids have been reported as potential biomarkers of kidney function, aging, and diabetes [50]. Therefore, it is also important to test the applicability of our method in other tissues.

## 4. Materials and Methods

### 4.1. Reagents

The standard compounds of l-Ala, l-Asn, l-Asp, l-Leu, l-Met, l-Pro, l-Ser, d-Asp, and d-Ser were purchased from Nacalai Tesque (Kyoto, Japan). l-Gln, l-Glu, l-Phe, d-Ala, d-Gln, d-Glu, d-Leu, d-Met, d-Phe, d-Pro, and l-FDLA were purchased from Tokyo Chemical Industry Co. Ltd. (Tokyo, Japan). d-Asn, LC/MS-grade ultrapure water and methanol, and ammonium formate were purchased from Wako Pure Chemical Industries Ltd. (Osaka, Japan).

### 4.2. Animals

Male C57BL/6N mice were obtained from Japan SLC Inc. (Hamamatsu, Japan) and were kept under a controlled temperature and standard light conditions (a 12:12 h light–dark cycle). They were fed a standard chow diet (CLEA Japan Inc., Tokyo, Japan) with free access to water for 3 or 24 months. All the animal experiments were approved by the Animal Experiment Committee of University of Toyama (Approval number A2017MED-11) and were performed in accordance with the Guidelines for the Care and Use of Laboratory Animals at the University of Toyama, which are based on international policies.

### 4.3. Metabolite Extraction from Animal Tissues

Metabolite extraction was described elsewhere [51]. Briefly, cerebellum, cortex, hippocampus, and thalamus were excised from 3- and 24-month-old mice. The tissues were immediately frozen in liquid nitrogen and kept at −80 °C until use. Wet tissues weighing 30 mg were ground in 1 mL of ice-cold 50% methanol—50% water by using a multibeads shocker (Yasui Kikai, Osaka, Japan) under optimal conditions. The lysate was centrifuged, and the supernatant was collected into a new tube. Then, the same volume of chloroform was added to the supernatant. The mixture was centrifuged at 13,000× *g* for 10 min at 4 °C. The separated upper aqueous phase was transferred into a new tube and the same procedure was repeated one more time. Finally, the aqueous phase was dried and reconstituted in LC/MS-grade water.

### 4.4. Derivatization of Amino Acids

Standard amino acid compounds were derivatized by l-FDLA before the separation by HPLC. To derivatize amino acid compounds, 50 µL of the standard solution were mixed with 10 µL of 200 mM sodium bicarbonate and 10 µL of 1% l-FDLA in acetone. The mixture was incubated at 40 °C for 1 h. After returning to room temperature, 930 µL of 50% methanol—50% water was added to the mixture. Subsequently, 10 µL of the solution was mixed with 490 µL of water followed by filtration using a 0.45 µm Milex filter unit (Merck Millipore, Burlington, VT, USA). To derivatize tissue samples, 50 µL of the reconstituted tissue samples mentioned above were processed as same as standard compounds. During these procedures, we keep samples in dark conditions.

### 4.5. LC/MS/MS Condition

Chromatographic analysis was performed by using an Agilent 6460 Triple Quad mass spectrometer that was coupled with an Agilent 1290 HPLC system. The detection of metabolites was conducted using positive ESI and multiple reaction monitoring (MRM) mode. The mass spectrometer settings were described previously [52]. To optimize MRM settings, standard compounds were prepared at a concentration of 1 µM and 10 µL of the solution was isocratically injected into the mass spectrometer having 50% of the mobile phase A (5 mM ammonium formate in water) and 50% of the mobile phase B (100% methanol) at a flow rate of 150 µL/min. [M+H]^+^ ion was selected as a precursor ion for all amino acids. The collision energy to produce product ions was selected by checking the maximum intensity for each amino acid. The optimized MRM settings for the derivatized amino acids are listed in Table 1. The HPLC separation of the amino acids was performed by using an MG3 column (2.0 × 150 mm, particle size of 3 µm, Osaka Soda, Osaka, Japan), having a gradient of mobile phase A (5 mM ammonium formate in water) and mobile phase B (100% of methanol) at a flow rate of 150 µL/min. The programmed mobile phase gradient was as follows: 0–10 min, 20–80% B; 10–15 min, 80% B; 15–15.01 min, 80–20% B. The column was equilibrated prior to sample injection, and the temperature of column oven was set at 40 °C.

### 4.6. Quantification of Amino Acids

To generate a standard curve of l- and d-amino acids, the standard compounds were diluted in water at concentrations adjusted for tissue contents. After derivatization, 10 µL of the standard solution or tissue solution was separated and detected using the LC/MS/MS system. Each chromatographic area was integrated to calculate the amount of the compounds using the Mass Hunter Quantitative analysis software (Agilent Technologies, Santa Clara, CA, USA).

### 4.7. Statistical Analysis

The differences between the young and old tissues were analyzed by using an unpaired Student’s *t*-test.

## 5. Conclusions

In conclusion, the absolute quantification of d-amino acid levels using LC/MS/MS combined with l-FDLA pre-derivatization is a useful and easy method for crude biological samples. Using this method, we found an alteration in levels of several d-amino acids during aging. Our method is more sensitive, at lease for several d-amino acids, compared with the methods previously reported. It is important to improve the sensitivity and to detect very low concentration d-amino acids in various tissues.

## Figures and Tables

**Table 1:** Multiple reaction monitoring (MRM) settings and retention time for the derivatized l- and d-amino acids. Standard l- and d-amino acids (AA) were derivatized with l-FDLA and measured by LC/MS/MS with positive ESI mode. The monoisotopic mass of standard amino acids before and after the derivatization (Da), precursor ion (*m*/*z*), product ion (*m*/*z*), collision energy (eV), and retention time (min) were shown.

	Monoisotopic Mass					Retention Time	
	Before Derivatization	After Derivatization	Precursor Ion (*m*/*z*)	Product Ion (*m*/*z*)	Collision Energy (eV)	l-AA (min)	d-AA (min)
Ala	89.0477	383.1441	384	339	6	17.0	18.9
Asn	132.0535	426.1499	427	382	8	16.0	17.0
Asp	133.0375	427.1339	428	383	6	14.0	14.9
Gln	146.0691	440.1656	441	334	10	16.7	17.3
Glu	147.0532	441.1496	442	397	8	15.2	16.0
Leu	131.0946	425.1910	426	380	4	17.9	22.4
Met	149.0510	443.1475	444	354	8	17.4	20.2
Phe	165.0790	459.1754	460	415	8	17.7	21.0
Pro	115.0633	409.1597	410	365	10	16.8	18.5
Ser	105.0426	399.1390	400	355	6	16.4	17.1

**Table 2:** Absolute quantification of l- and d-amino acids in various brain regions from young and old mice. Metabolites were extracted from the cerebellum, cortex, hippocampus, and thalamus in young (3-month-old) and old (24-month-old) mice. After the derivatization with F-FDLA, the levels of l- and d-Asp, Asn, Ser, and Phe were absolutely quantified by LC/MS/MS. Amounts of l- and d-amino acids were calculated by using the standard curves obtained in Figure 5. The data is represented as means ± SD from young (*n* = 5) and old (*n* = 8) animals.

		Young	Old	
l-Asn (µmol/g tissue)	Cerebellum	0.107 ± 0.018	0.105 ± 0.024	n.s.
	Cortex	0.135 ± 0.038	0.131 ± 0.016	n.s.
	Hippocampus	0.150 ± 0.015	0.113 ± 0.019	*p* < 0.05
	Thalamus	0.130 ± 0.025	0.122 ± 0.022	n.s.
d-Asn (µmol/g tissue)	Cerebellum	0.000725 ± 0.000111	0.00100 ± 0.00047	n.s.
	Cortex	0.000859 ± 0.000246	0.00103 ± 0.00024	n.s.
	Hippocampus	0.00107 ± 0.00006	0.000893 ± 0.000230	n.s.
	Thalamus	0.000625 ± 0.000136	0.000620 ± 0.000222	n.s.
l-Asp (µmol/g tissue)	Cerebellum	11.3 ± 2.3	9.39 ± 2.28	n.s.
	Cortex	7.02 ± 2.04	7.79 ± 0.44	n.s.
	Hippocampus	9.54 ± 1.04	7.37 ± 1.24	*p* < 0.05
	Thalamus	7.88 ± 0.74	6.73 ± 1.51	n.s.
d-Asp (µmol/g tissue)	Cerebellum	0.0126 ± 0.0053	0.0147 ± 0.0043	n.s.
	Cortex	0.0675 ± 0.0253	0.0608 ± 0.0062	n.s.
	Hippocampus	0.0825 ± 0.0130	0.0560 ± 0.0121	*p* < 0.05
	Thalamus	0.0422 ± 0.0195	0.0311 ± 0.0060	n.s.
l-Phe (µmol/g tissue)	Cerebellum	0.107 ± 0.009	0.119 ± 0.034	n.s.
	Cortex	0.0996 ± 0.0231	0.112 ± 0.019	n.s.
	Hippocampus	0.129 ± 0.012	0.127 ± 0.026	n.s.
	Thalamus	0.0966 ± 0.0212	0.101 ± 0.032	n.s.
d-Phe (µmol/g tissue)	Cerebellum	0.287 ± 0.096	0.221 ± 0.041	n.s.
	Cortex	0.246 ± 0.059	0.201 ± 0.057	n.s.
	Hippocampus	0.207 ± 0.069	0.209 ± 0.057	n.s.
	Thalamus	0.211 ± 0.015	0.241 ± 0.015	*p* < 0.05
l-Ser (µmol/g tissue)	Cerebellum	0.983 ± 0.114	1.06 ± 0.29	n.s.
	Cortex	1.31 ± 0.34	1.30 ± 0.07	n.s.
	Hippocampus	1.56 ± 0.09	1.32 ± 0.18	*p* < 0.05
	Thalamus	0.896 ± 0.235	0.819 ± 0.139	n.s.
d-Ser (µmol/g tissue)	Cerebellum	0.0285 ± 0.0158	0.0211 ± 0.0080	n.s.
	Cortex	0.609 ± 0.156	0.580 ± 0.048	n.s.
	Hippocampus	0.646 ± 0.041	0.526 ± 0.060	*p* < 0.01
	Thalamus	0.313 ± 0.109	0.279 ± 0.036	n.s.

n.s.: not significant.

## Data Availability

The data presented in this study are available in the article.
